# Rubella Epidemic Strain, Greece, 1999

**DOI:** 10.3201/eid1009.040117

**Published:** 2004-09

**Authors:** Anna Papa, Georgia Gioula, Antonis Antoniadis, Vassiliki Kyriazopoulou-Dalaina

**Affiliations:** * Aristotle University of Thessaloniki, Thessaloniki, Greece

**Keywords:** letter, rubella, Greece, epidemic

**To the Editor:** A recent extensive study on global distribution of rubella virus genotypes by Zheng et al. (*1*) showed that most of the isolates tested were rubella genotype I (RGI) and that subgenotypes within RGI were apparent. Of these subgenotypes, three were currently active, one in the United States and Latin America, one in China represented by two specimens, and one international subgenotype that originated in Asia and spread to Europe and North America. More RGI subgenotypes, which have not yet been identified in specimen collections, may be currently active. In Zheng, et al. the distribution of rubella subgenotypes is shown; Greece is one of the four European countries where only RGI viruses were found. This letter provides more information about rubella in Greece and the strain that was responsible for the 1999 epidemic there.

Rubella virus is endemic in Greece. Vaccination in the private sector only was introduced in 1981 with a monovalent rubella vaccine. In 1989, a single dose of the measles-mumps-rubella (MMR) vaccine was introduced in the national vaccination program for 15-month-old infants. Because of the rubella outbreak of 1993, the vaccination policy changed. In 1997, a second vaccination was recommended for 11- to 12-year-old children. However, another major rubella epidemic occurred in 1999, beginning in late December 1998 and lasting until May 1999, with a peak in the number of cases in January. During this period, 1,438 rubella cases were reported throughout Greece; 765 were in the northern part of the country. In previous rubella epidemics in Greece, children were most affected. However, in the 1999 epidemic, a higher incidence rate was observed mostly among 15- to 19-year-old persons (*2*). During this epidemic, four cases of congenital rubella syndrome were reported. Because of this epidemic, the vaccination policy was revised. The new policy consists of two doses of the MMR vaccine, one to be given at 15 months of age and another to be given at 4–6 years of age (*3*).

During the 1999 epidemic, oral samples were collected from patients within a week of the onset of symptoms. Most of the samples were sent to Colindale, London, for testing; a few of them were stored at –70°C in our laboratory at the School of Medicine, Aristotle University of Thessaloniki, Thessaloniki, Greece. In 1999, four universities in the United Kingdom reported cases of rubella infection. Greek students were attending all of these universities; the students had spent the Christmas holidays in Greece and then returned to the United Kingdom. The U.K. rubella strains were identical to those of the Greek epidemic strain (*4*). We amplified and sequenced a 143-bp segment of the E1 gene by using reverse transcription-nested polymerase chain reaction from the samples stored in our laboratory (*5*). All 10 samples tested contained very similar or identical sequences, so we used one of them as the epidemic rubella strain. Comparing the Greek strain (Thess1/GRE99, accession no. AY540614) with rubella sequences taken from GenBank, we found that it belonged to the international (1997–2000) rubella RGI subgenotype. Although the genome region tested was short, the Greek rubella strain was highly homologous to the strains isolated from Germany in 1999 (G432/GER99, accession no. AF551761) and from Italy in 1997 (6423/PV/ITA97, accession no. AY161374). However, the Greek strain had a genetic difference of approximately 5% from strains isolated from Italy (4844/ITA93, accession no. AY161364), the United Kingdom (DNY/UNK93, accession no. AF039131) in 1993, and Germany (D075/GER92, accession no. AF039118) in 1992 and (G696/GER98, accession no. AY326342) in 1998. We also found that the Greek strain differed by 6% from the RA27/3 vaccine strain, predominantly used for RGI.

Zheng et al. (*1*) showed that the RGI-ITA97 genotype strain was closely related to a genotypic group of viruses isolated from the eastern United States and two Caribbean countries from 1999 to 2001 (*6*). Results from our phylogenetic analysis showed that Thess1/GRE99, as well as strains G432/GER99 and 6423/PV/ITA97 (and the United Kingdom 1999 strains), were clustering with strains FRI/BAH97 (accession no. AY326359), isolated in 1997 from the Bahamas, and DES/MB-CAN97 (AY326358), isolated in 1997 from Manitoba, Canada (which also belong to the international 1997–2000 RGI genotype). These findings indicated that this rubella strain was circulating in both Europe and North America at least as early as 1997.

Recent data indicate that internationally circulating rubella viruses exist, even when vaccination programs are conducted. Comprehensive specimen collection and genotypic analysis are necessary to identify and track the emergence and spread of such genotypes.
